# Correction: Liu, W. et al. Error Overboundings of KF-Based IMU/GNSS Integrated System Against IMU Faults. *Sensors* 2019, *19*, 4912

**DOI:** 10.3390/s20216274

**Published:** 2020-11-04

**Authors:** Wei Liu, Dan Song, Zhipeng Wang, Kun Fang

**Affiliations:** School of Electronic and Information Engineering, Beihang University, Beijing 100191, China; lw1014hjh@163.com (W.L.); songdan0207@buaa.edu.cn (D.S.); fangkun@buaa.edu.cn (K.F.)

To clearly highlight the differences between the error overboundings of error-state EKFs and full-state EKFs. The authors wish to make the following corrections to this paper [1]:

## 1. Changes in Title

The title is changed to:

Comparative Analysis Between Error-State and Full-State Error Estimation for KF-Based IMU/GNSS Integration Against IMU Faults

## 2. Changes in Abstract

Lines 2–9, the sentences “this paper studies the error overboundings of the state estimation of the extended Kalman filter (EKF) in a tightly coupled IMU/global navigation satellite system (GNSS) integrated architecture under the IMU fault condition, which can be used to assure the integrity of the UAV navigation system. The error overboundings of the error-state inertial navigation equations based EKF (error-state EKF) are obtained according to the IMU faults propagation derivation, which can be expressed as a sum of the terms related to the EKF innovation, the estimated bias, and the remaining position error. It presents the same expression with the error overbounding of the full-state inertial navigation equations based EKF (full-state EKF).” should be changed to “this paper provides an analysis of the error overboundings of position estimation in a tightly coupled IMU/global navigation satellite system (GNSS) integrated architecture under the IMU fault conditions using an error-state EKF-based approach and provides a comparison to a recently published EKF-based full state method.”

## 3. Changes in Section 1 Introduction

There are two mistakes in the use of gender pronouns in paragraph 5 line 2 and line 4: 

In paragraph 5 line 2, the sentence “Lee proposed an integrity assurance mechanism for an EKF-based IMU/GNSS integrated system against IMU faults” should be changed to “Lee proposed an integrity assurance mechanism for an EKF-based IMU/GNSS integrated system against IMU faults [18]”. The sentence “He calculated the PLs using the EKF innovations and additional uncertain noise boundary terms” should be changed to “She calculated the PLs using the EKF innovations and additional uncertain noise boundary terms”. In paragraph 5 line 4, the sentence “In his study,” should be changed to “In her study,”.

In paragraph 5 line 6, the sentences “However, only the velocity update equation was used as an example to explain how the IMU faults propagate in the EKF. Since the inertial navigation equation contains three update equations of the position, velocity, and attitude, and the faults have different expressions in the three update equations. The attitude and position update equations also need to be considered for analyzing the IMU faults propagation process.” should be changed to “Moreover, the velocity update equation was used as an example to explain how the IMU faults propagate in the EKF”.

In paragraph 6 line 1, the sentence “In our study, the error-state inertial navigation equations” should be changed to “In our study, different from the method published by Lee et al. [18], the error-state inertial navigation equations”. In paragraph 6 line 4, the sentence “than the full-state inertial navigation equations” should be changed to “than the full-state inertial navigation equations [18–20]”. In paragraph 6 line 5, the sentence “The error overboundings against the IMU faults for the EKF state estimation using the error-state inertial navigation equation is derived in this paper.” should be changed to “While being inspired by the previous published research in this area [16–20], the error overboundings against the IMU faults for the EKF state estimation using the error-state inertial navigation equation is derived in this paper”.

In paragraph 6 line 9, we added:

“After using the inertial navigation error equation, the magnitude of each dimension of the state vector keep close or consistent, and the occurrence of singular matrices in the filtering calculation process is avoided, which will shorten the filtering time, improve the converge speed, and increase the calculation efficiency.”

## 4. Changes in Section 3 Error Overboundings Against IMU Faults

We added the sentences “Different from the full-state EKF, the error-state EKF has no input of the control quantity during the entire filtering process. Compared with the full-state EKF, the error-state EKF keeps the magnitude of each dimension of the state vector close or consistent, which avoids the occurrence of singular matrices in the filtering calculation process, reduces the convergence time and improves the calculation efficiency.” before the introductory paragraph. 

## 5. Changes in Section 3.1 IMU Faults Propagation in the Error-State EKF

In paragraph 1 line 2, we added: “While following a similar approach, it is worth mentioning that the method derived here is a different approach from the work published by Lee et al [18] where error states instead of full navigation states are considered.”

The sentence “The discrete form of Equation (16) is shown in Equation (17):” should be changed to “The discrete form of Equation (16) is shown in Equation (17) [18]:”. The sentence “Substituting Equation (19) into Equation (2), the measurement update state under the IMU faults condition at epoch k is” should be changed to “Substituting Equation (19) into Equation (2), the measurement update state under the IMU faults condition at epoch k is [18]:”. The sentence “To simplify Equation (20), we introduce a matrix $L_{k}{}^{’}$ as shown in Equation (21):” should be changed to “To simplify Equation (20), a matrix $L_{k}{}^{’}$ is introduced as Equation (21) [18]:”.

## 6. Changes in Section 3.2 EKF State Error Caused by IMU Faults

We deleted the sentence “The PL against an IMU gyroscope and accelerometer fault should be changed to formulated to overbound the state error with the integrity risk requirement.” and changed the following sentences:

The true state can be defined as shown in Equation (23):

The sentence “The state error propagation between two adjacent epochs can be derived as follows:” should be changed to “According to the previous study [18], the state error propagation between two adjacent epochs can be derived as follows:”.

We deleted the sentence “As shown in Equation (27), the state error vector at epoch k can be expressed as a function of the previous state error vector, a state fault vector, a measurement noise vector, and a process noise vector, indicating that the derived state error vector affected by the IMU faults is recursive”.

## 7. Changes in Section 3.3 Error Overboundings

We deleted the first paragraph, and derivation process and text after Equation (28), and added the following sentences:

“As Equations (27) and (28) show, the final EKF state error and EKF innovation using the error-state EKF were the same as that obtained by the full-state EKF in [18]. Based on the method proposed in [18] for the full-state EKF, the error overboundings against the IMU faults for the error-state EKF can be expressed as Equation (29), which was originally derived in [18] for the full-state EKF.

(29) $$PL_{error-overboundings}=-V_{k,p}{\vec{\gamma }}_{k}\pm K_{md,IMU}\left\{\sigma_{V_{k,p}{\vec{v}}_{k},v}+\sigma_{K_{k,p}{}^{’}{\vec{v}}_{k},v}\right\}.$$

As we expected, the final error overboundings equation (Equation (29)) can be expressed in an identical manner to those derived based on the full-state EKF in the previous study [18].”

## 8. Changes in Section 4. Simulation and Analysis

We added a paragraph at the end of the Section 4: “To sum up, error-state EKF-based method and full-state EKF-based method are consistent in final equation expression and overbounding results of the error overboundings. However, the qualitative analysis result shows that error-state EKF-based method has a higher calculation efficiency and faster convergence speed, and the quantitative comparison result in Table 2 shows error-state EKF-based method has shorter filtering time.”

## 9. Changes in Section 5. Conclusions

In the beginning of this part, we added:

“A real-time full-state EKF vertical protection level (VPL) method against IMU sensor faults to assure navigation integrity is proposed in [18]. Based on the method, the final error overbounding equation against the IMU faults for the error-state EKF estimation can be expressed in an identical manner.”

## 10. Changes in Section Appendix C. IMU Faults Propagation in the Full-State EKF

After the first paragraph, we added: 

“The velocity update equation is used as an example to explain the IMU faults propagation in the full-state EKF in [18]. In this appendix, refer to the methods proposed in [18], the detailed derivation of error propagation using full-state EKF in position, velocity and attitude equations are given.”

The sentence “The discrete form of Equation (A34) is shown in Equation (A35):” should be changed to “The discrete form of Equation (A34) is shown in Equation (A35) [18]:”. The sentence “By substituting Equation (A37) into Equation (A2), the measurement update state under the IMU faults condition at epoch k is” should be changed to “By substituting Equation (A37) into Equation (A2), the measurement update state under the IMU faults condition at epoch k is [18]:”. The sentence “To simplify Equation (A38), a matrix $L_{k}{}^{’}$ is introduced as shown in Equation (A39):” should be changed to “To simplify Equation (A38), a matrix $L_{k}{}^{’}$ is introduced as shown in Equation (A39) [18]:”.

We deleted the sentence “The PL against an IMU gyroscope and accelerometer fault should be changed to formulated to overbound the state error with the integrity risk requirement”.

The sentence “where ${\vec{X}}_{k}$ represents the true state as shown in Equation (A42):” should be changed to “where ${\vec{X}}_{k}$ represents the true state as shown in Equation (A42) [18]:”.

The sentence “Thus, the state error propagation between two adjacent epochs can be derived as follows:” should be changed to “Thus, according to the previous study [18], the state error propagation between two adjacent epochs can be derived as follows:”.

We deleted the sentence “As shown in Equation (A46), the state error vector at epoch k can be expressed as a function of the previous state error vector, a state fault vector, a measurement noise vector, and a process noise vector, indicating that the derived state error vector affected by the IMU faults is recursive”.

## 11. Changes in Section References

The reference 18: “18. Lee, J.L.; Kim, M.; Lee, J.Y.; Pullen, S. Integrity Assurance of Kalman-Filter Based GNSS/IMU Integrated Systems Against IMU Faults for UAV Applications; ION GNSS: Miami, FL, USA, 2018; pp. 2484–2500.” should be changed to “18. Lee, J.; Kim, M.; Lee, J.; Pullen, S. Integrity Assurance of Kalman-Filter Based GNSS/IMU Integrated Systems Against IMU Faults for UAV Applications; ION GNSS: Miami, FL, USA, 2018; pp. 2484–2500”.

These changes have no material impact on the conclusions of our paper. The authors would like to apologize for any inconvenience caused to the readers by these changes.
